# Bile acid homeostasis controls CAR signaling pathways in mouse testis through FXRalpha

**DOI:** 10.1038/srep42182

**Published:** 2017-02-09

**Authors:** Emmanuelle Martinot, Marine Baptissart, Aurélie Véga, Lauriane Sèdes, Betty Rouaisnel, Fred Vaz, Jean-Paul Saru, Angélique de Haze, Silvère Baron, Françoise Caira, Claude Beaudoin, David H. Volle

**Affiliations:** 1 INSERM U 1103, Génétique Reproduction et Développement (GReD), F-63170 Aubière, France; 2 Université Clermont Auvergne, GReD, F-63000 Clermont-Ferrand, F-63170 Aubière, France; 3 CNRS, UMR 6293, GReD, F-63170 Aubière, France; 4 Centre de Recherche en Nutrition Humaine d’Auvergne, F-63000 Clermont-Ferrand, France; 5 Academic Medical Center, University of Amsterdam, Lab. Genetic Metabolic Diseases, F0-224, P.O. Box 22700, 1100 DE Amsterdam, The Netherlands

## Abstract

Bile acids (BAs) are molecules with endocrine activities controlling several physiological functions such as immunity, glucose homeostasis, testicular physiology and male fertility. The role of the nuclear BA receptor FXRα in the control of BA homeostasis has been well characterized. The present study shows that testis synthetize BAs. We demonstrate that mice invalidated for the gene encoding FXRα have altered BA homeostasis in both liver and testis. In the absence of FXRα, BA exposure differently alters hepatic and testicular expression of genes involved in BA synthesis. Interestingly, Fxrα-/- males fed a diet supplemented with BAs show alterations of testicular physiology and sperm production. This phenotype was correlated with the altered testicular BA homeostasis and the production of intermediate metabolites of BAs which led to the modulation of CAR signaling pathways within the testis. The role of the CAR signaling pathways within testis was validated using specific CAR agonist (TCPOBOP) and inverse agonist (androstanol) that respectively inhibited or reproduced the phenotype observed in Fxrα-/- males fed BA-diet. These data open interesting perspectives to better define how BA homeostasis contributes to physiological or pathophysiological conditions *via* the modulation of CAR activity.

Bile acid (BAs) levels are increased during liver diseases^1^,^2^ and have been defined as the most consistent change in the early phase of several liver diseases out of 1900 metabolites screened in the plasma, urine and liver^3^. BAs are molecules with endocrine activities controlling several physiological functions such as immunity, glucose homeostasis and energy metabolism^4^ as well as male sexual maturation and fertility^5^,^6^. The altered BA homeostasis appears to be involved in the etiology of several diseases and thus needs to be strictly regulated.

The BA nuclear receptor Farnesoid-X-Receptor-α (FXRα; NR1H4) is a key actor of BA homeostasis *via* synergistic pathways within the liver and intestine. Indeed, FXRα activation and/or inhibition modulate the composition of BA-pool. At the molecular and cellular levels, in the liver, FXRα represses BA synthesis through a cascade involving the *small heterodimer partner receptor* (SHP; NR0B2) and the *liver receptor homolog-1* (LRH-1; NR5A2)^7^,^8^. This leads to the repression of the expression of *cytochrome P450 Cyp7a1*, the rate-limiting enzyme for the conversion of cholesterol into BAs. FXRα induces the expression of the *bile salt export pump (Bsep*) gene which is involved in the secretion of BAs^9^. In the ileum, FXRα controls the reabsorption of BA through the regulation of the organic solute transporters (OSTα and OSTβ)^9^. A major role of FXRα is consequent to its impact on the expression of *fibroblast growth factor* (FGF) Fgf15. This represses *Cytochrome (Cyp) Cyp7a1* expression in the liver *via* the FGFR4/βKlotho signaling pathway^10^. Recent data using tissue-specific Fxrα-null mice show that the intestinal FXRα-FGF15 pathway plays a more important role than the FXRα-SHP cascade^11^. The significance of these actions of FXRα was recently sustained by the fact that in human, mutations of the gene encoding FXRα have been identified as a cause of progressive familial intrahepatic cholestasis^12^. Thus, the impacts of the lack of FXRα activity on BA homeostasis in different tissues still need to be better defined.

Previous studies have shown that detectable levels of BAs are present in testis even in normal physiological condition^5^,^13^. It is thus interesting to question whether 1/these BAs come from plasma only or could be synthetized by the testis; 2/how BA homeostasis is regulated in different organs like liver and testis and 3/the potential involvement of FXRα in these regulations. In that line, the present study shows that testis could also synthetize BAs. Moreover, the mice invalidated for the gene encoding FXRα have alterations of bile acid homeostasis in liver and testis. The exposure to BAs alters differently the hepatic and the testicular expression of genes involved in BA synthesis in wild type (Wt) and *Fxrα*−/− males. We show that in the context of FXRα deficiency, BA exposure results in a strong alteration of testis physiology and sperm production *via* CAR signaling pathways. Indeed, the testicular altered BA pool composition and the production of intermediate metabolites of BA led to the modulation of constitutive-androstane-receptor (CAR; NR1I3) signaling pathways within the testis. The involvement of CAR is supported by our data showing that within the testis, exposure to a specific CAR agonist or inverse agonist respectively counteracted or reproduced the phenotype observed in Fxrα−/− mice fed BA-diet.

## Results

### Testis produces bile acids

**Figure 1:**
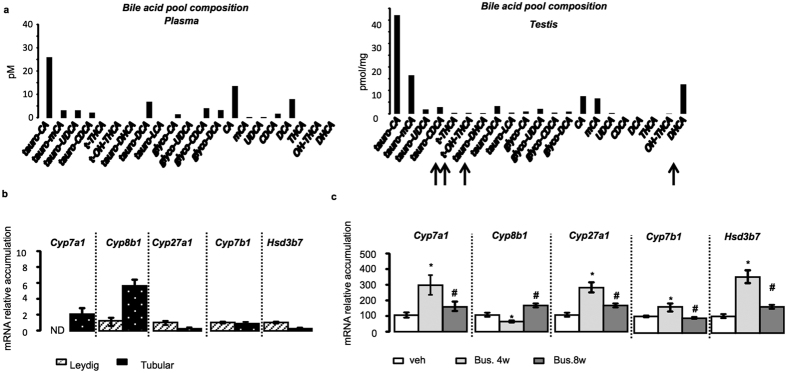
Testis cells express genes involved in bile acid synthesis. (**a**) Plasma and testicular bile acid pool compositions in wild-type (n = 5 per group). (**b**) mRNA expression of *Cyp7a1, Cyp8b1, Cyp27a1, Cyp7b1* and H*sd3b7* in purified Leydig cells and tubular compartment of testis normalized to *β*-*actin* mRNA levels in wild-type mice (n = 6–10 per group). (**c**) Testicular mRNA accumulation of *Cyp7a1, Cyp8b1, Cyp27a1, Cyp7b1* and H*sd3b7* normalized to *β-actin* mRNA levels in whole testes of C57BL/6J mice treated with busulfan (20 mg/kg, one injection IP) at T0, 4, or 8 weeks (n = 8 per group). Data are expressed as means ± standard error of the mean. *Denotes significant difference from the T0 time point; #denotes significant difference from the 4-week time point (*P* < 0.05)%. In all panels, data are expressed as means ± standard error of the mean. Statistical analysis: *P < 0.05. *Denotes

Detectable levels of bile acids have been measured in the testis of mice in normal physiological conditions^5^. Interestingly, a careful analysis showed differences between plasma and testicular BA pool compositions (Fig. 1a). The synthesis of BAs by the testis, *in vivo*, was sustained by the detection of dihydroxycholestanoic acid (DHCA) and Trihydroxycholestanoic acid (THCA) within the testis whereas such intermediate metabolites were not detected within the plasma BA pool (Fig. 1a). To ensure the possibility that testis can produce BAs we analyzed the expression of key enzymes of BA synthesis pathways in different fractions of testicular cell types (interstitial versus tubular compartment) (Fig. 1b). All tested enzymes are expressed in testis. *Cyp8b1 (Cytochrome P450 8b1), Cyp27a1, Cyp7b1* and *Hsd3b7* (Hydroxysteroid dehydrogenase) were detected both in the tubular compartment and in the Leydig cells of the testis (Fig. 1b). *Cyp7a1* was not detected in samples from purified Leydig cells (Fig. 1b). We also analyzed their expression pattern in a classical model of transitory germ cell loss using exposure to busulfan^5^. Compared to specific markers of Leydig (luteinizing hormone/choriogonadotropin receptor-*Lhcgr*), Sertoli (follicle-stimulating hormone receptor-*Fshr*), and germ cells at different steps of spermatogenesis (*Nanog, Oct3/4, G9a, Ccna1* and *Smad6)* (Fig. S1a), the observed expression patterns indicated that *in vivo Cyp7a1, Cyp27a1, Cyp7b1* and *Hsd3b7* are mainly expressed in somatic cells or spermatogonia; whereas *Cyp8b1* showed ubiquitous expression pattern (Fig. 1c).

**Figure 2:**
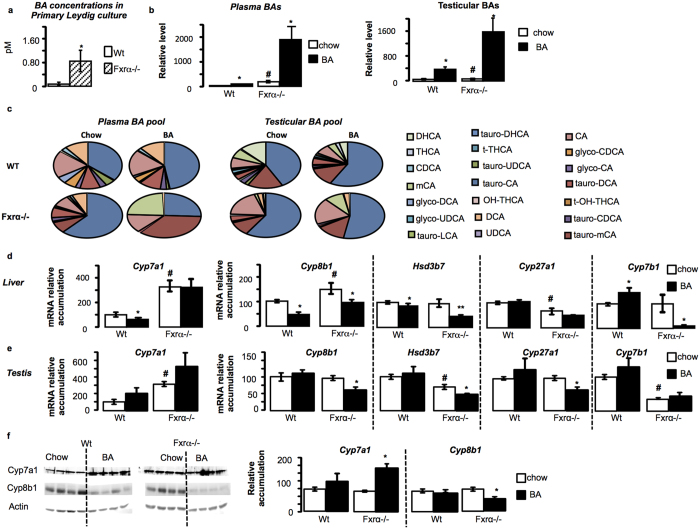
Fxrα deficiency alters BA homeostasis in liver and testis. (**a**) BA levels produced by primary culture of wild-type (Wt) or Fxrα−/− Leydig cells 24 hours after serum starvation (n = 9 per genotype). (**b**) Plasma and testicular total bile acid levels in Wt and Fxrα−/− fed either chow-diet or BA-diet for 2 weeks (n = 5 per genotype). (**c**) Composition of plasma and testicular bile acid pool composition in wild-type and Fxrα−/− mice fed a control or BA-diet for 2 weeks (n = 5 per group). (**d**) Liver mRNA accumulation of *Cyp7a1, Cyp8b1*, H*sd3b7, Cyp27a1* and *Cyp7b1* normalized to *β*-*actin* mRNA levels in wild-type and Fxrα−/− adult mice fed a control or BA-diet (n = 6–10 per group). (**e**) Testicular mRNA accumulation of *Cyp7a1, Cyp8b1*, H*sd3b7, Cyp27a1* and *Cyp7b1* normalized to *β*-*actin* mRNA levels in wild-type and Fxrα−/− adult mice fed a control or BA-diet (n = 6–10 per group). (**f**) Testicular protein accumulation of Cyp7a1 and Cyp8b1 normalized to *Actin* levels in wild-type and Fxrα−/− adult mice fed a control or BA-diet (n = 6 per group). In all panels, wild-type control diet group was arbitrarily fixed at 100%. In all panels, data are expressed as means ± standard error of the mean. Statistical analysis: *P < 0.05. *Denotes significant difference between same genotype under different diets; #denotes significant difference between differents genotype under same diet exposure.

Comparing whole testis mRNA accumulations to liver, it appears that the mRNA levels of genes involved in BA synthesis were much lower expressed in testis (Fig. S1b). As supported by busulfan experiments, the low expression levels in testis could reflect the fact that these genes are mainly expressed in a low number of cells such as Sertoli and/or Leydig cells and/or spermatogonia which represent only 5 to 15% of the testis. Primary culture of Leydig cells was performed to ensure that the expression of these enzymes have significant relevance within the testis. Data demonstrate that these cells were able to synthesize BAs (Fig. 2a).

### FXRα is a main regulator of testicular BA homeostasis

It was previously reported that FXRα deficiency has major impact on liver expression of genes involved in BA synthesis^14^. In the present study, and consistently with previously published studies, the *Fxrα*−/− males showed higher levels and altered pool composition of plasma BAs compared to wild-type mice (Fig. 2b,c). This was associated with altered expression of genes involved in BA synthesis. In the liver of Fxrα−/− males, there was an upregulation of *Cyp7a1, Cyp8b1* and a downregulation of *Cyp27a1* mRNA accumulation (Fig. 2d); whereas the mRNA accumulations of *Hsd3b7* and *Cyp7b1* were not affected compared to Wt (Fig. 2d).

The testicular BA levels and pool compositions were also modified by genotype (Wt versus Fxrα−/−) (Fig. 2b,c). Such higher BA levels were observed in primary culture of Fxrα−/− Leydig cells compared to Wt. This suggests that Fxrα−/− testes have higher capacity to generate BAs such as in liver (Fig. 2a).

At the molecular level, the alterations were different within the testis. Indeed, the *Fxrα* deficiency led to an increased mRNA accumulation of *Cyp7a1*, a downregulation of *Hsd3b7* and *Cyp7b1* mRNA accumulations compared to Wt. No impact was observed on the testicular expression of *Cyp8b1* and *Cyp27a1* between genotypes (Fig. 2e).

These data suggest that FXRα might be a key intra-testicular regulator of BA homeostasis. To better decipher its involvement, wild type and Fxrα−/− males were fed a BA-diet (Fig. 2). The plasma and testicular BA levels and pool compositions were differently affected by the exposure to BA-diet (Fig. 2b,c).

In liver of Wt males, the mRNA accumulations of *Cyp7a1* and *Cyp8b1* were decreased whereas the one of *Cyp7b1* was increased in response to BA-diet (Fig. 2d). In Fxrα−/− mice, *Cyp7a1 and Cyp27a1* mRNA accumulations were not affected by the BA-diet (Fig. 2d). In contrast, the mRNA accumulation of *Cyp8b1, Hsd3b7* and *Cyp7b1* were downregulated in Fxrα−/− males fed a BA-diet compared to Fxrα−/− males treated with control-diet (Fig. 2d).

Within the testis, no difference was noticed in the *Wt* males depending on the diet (Fig. 2e). In contrast, the expression of *Cyp8b1, Hsd3b7 and Cyp27a1* were decreased by the BA-diet in Fxrα−/− mice (Fig. 2e).

These results were validated at the protein level at least using Cyp7a1 and Cyp8b1 antibodies (Fig. 2f). Cyp8b1 was decreased in Fxrα−/− mice fed BA-diet whereas Cyp7a1 was increased in these mice.

### Dietary BA supplementation alters testicular physiology

**Figure 3:**
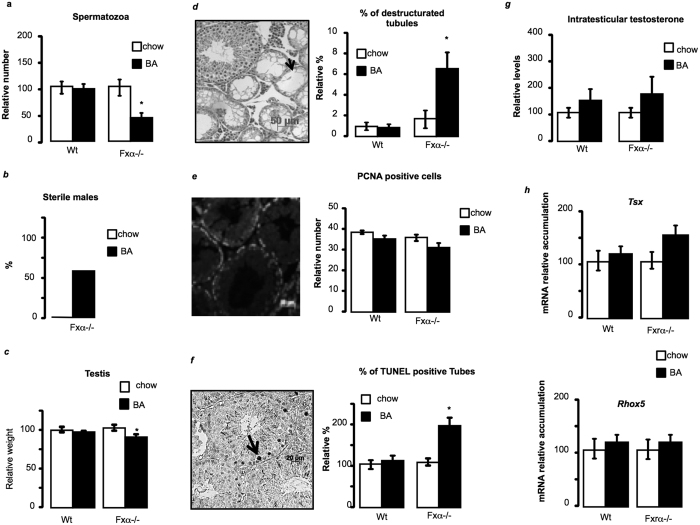
BA-diet induces male hypofertility associated with testicular defects in Fxrα−/− males. (**a**) Spermatozoa count in the heads of the epidydimis of wild-type and *Fxrα*−/−males exposed during 1 month to control or BA-diets (n = 10–20 per group). Control diet groups were arbitrarily fixed at 100%. (**b**) Relative percentage of sterile males; each male was bred with 2 C57Bl/6J females to analyze their reproductive capacity (n = 10–15 per group). (**c**) Relative testis weight normalized to body weight in wild-type and Fxrα−/− mice fed a control or BA-diet for 1 month (n = 10–20 per group). Control diet groups were arbitrarily fixed at 100%. (**d**) Representative micrograph of H&E-stained testis of Fxrα−/− male fed a BA-diet for 1 month. Arrows indicate tubes with complete loss of germ cells. Quantification of the number of completely destructured tubules per 100 tubules (n = 10–20 per group). (**e**) Germ cell proliferation in wild-type and Fxrα−/− mice exposed to control or BA-diets analyzed by PCNA staining. The number of PCNA-positive cells is indicated as the number of positive cells per 100 seminiferous tubules (n = 5–10 per group). (**f** ) Apoptosis in wild-type and Fxrα−/− mice exposed to control or BA-diets (n = 10–20 per group) analyzed by TUNEL staining. For the quantification of TUNEL analyses, the number of tubules with TUNEL-positive cells is indicated as the number of positive tubes per 100 seminiferous tubules (n = 10–20). Control-diet–treated mice were arbitrarily fixed at 100% for each genotype. (**g**) Relative intra-testicular testosterone levels in *Wt* and *Fxrα*−/−mice fed a control or BA-diet for 1 month (n = 7–10 per group). (**h**) Testicular mRNA accumulation of *Tsx* and Rhox5 normalized to *β*-*actin* mRNA levels in wild-type and Fxrα−/− mice fed a control or BA-diet for 1 month (n = 6–10 per group). Control diet groups were arbitrarily fixed at 100% for each genotype. In all of the panels, data are expressed as the means ± standard error of the mean. Statistical analysis: *P < 0.05.

In order to decipher the impact of the modulation of BA homeostasis on the testicular physiology in the context of Fxrα−/− mice, we first defined the testicular phenotype of Fxrα−/− males fed with BA-diet. Fxrα−/− males exposed to BA showed a 50% decreased of sperm production compared to control diet group whereas no effect of BA-diet was found in wild-type males (Fig. 3a). In that line, the Fxrα−/− males showed decreased reproductive capacities following BA-diet exposure with approximately 60% of the Fxrα−/− males exposed to BA-diet becoming sterile (Fig. 3b). The exposure to BA-diet led to a decrease of testis weight in FXRα−/− mice. This suggests abnormalities in testicular physiology (Fig. 3c). A higher number of destructurated seminiferous tubules were observed in these mice compared to the control-diet group (Fig. 3d). Testicular histology of wild-type males was not affected (Fig. 3d). BA-diet did not affect germ cell proliferation process in *Wt* and *Fxrα*−/− males (Fig. 3e). In contrast, Fxrα−/− males showed an increased apoptotic rate of germ cells in response to BA-diet, whereas no impact was noticed in wild-type mice (Fig. 3f). Germ cell apoptosis is often associated with low androgen levels^15^. However, BA-diet had no impact on testicular testosterone levels in wild-type or Fxrα−/− males (Fig. 3g). Results were sustained by the lack of effect of BA-diet on the mRNA accumulation of androgen-dependent genes, such as *Testis specific X-linked gene (Tsx) or* Reproductive homeobox 5 (*Rhox5*) in both wild type and Fxrα−/− males (Fig. 3h). This suggests that the apoptotic process in Fxrα−/− males exposed to BAs is independent of the testicular endocrine function.

### The testicular abnormalities are not TGR5-dependent

**Figure 4:**
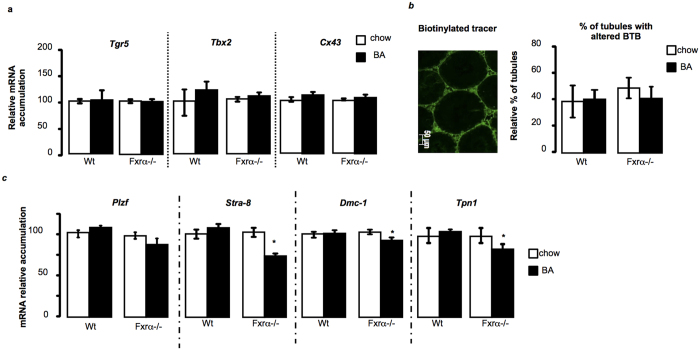
BA-diet induces male hypofertility in Fxrα−/− males in a Tgr5 independent manner. (**a**) Testicular mRNA accumulations of *Tgr5, Tbx2* and Cx43 normalized to *β*-*actin* mRNA levels in wild-type and Fxrα−/− mice fed a control or BA-diet for 1 month (n = 6 per group). Control diet groups were arbitrarily fixed at 100%. (**b**) Blood-testis-barrier integrity, as measured by the stained testes for EZ-link biotinylated. Representative micrographs of mice fed 1 month with a control diet or BA-diet. The original magnification was 100×. Quantification of the number of tubules with infiltration per 100 seminiferous tubules after 1 month of a control or BA-diet (n = 5 to 7 per group). (**c**) Testicular mRNA accumulation of *Plzf, Stra-8, Dmc-1*, and *Tpn1* normalized to *β*-*actin* mRNA levels in wild-type and Fxrα−/− mice fed a control or BA diet for 1 month (n = 6 per group). In all panels, control diet groups were arbitrarily fixed at 100%. In all panels, data are expressed as means ± standard error of the mean. Statistical analysis: *P < 0.05.

If previous study defined the major role of TGR5 in the pathophysiology of the adult testis in response to BA-diet^5^, several elements of the present study suggest that the observed effects in Fxrα−/− males in response to BA-diet are TGR5 independent. Here the apoptotic germ cells are pre-meiotic and/or meiotic germ cells and not post-meiotic ones (Fig. 3f). In addition, results showed no alteration of *Tgr5, T-box transcription factor-2 (Tbx2)* and *Connexin-43 (Cx43)* mRNA accumulations in wild type and Fxrα−/− males in response to BA-diet (Fig. 4a). This lack of effects on TGR5 pathway was supported by the fact that blood-testis-barrier was not altered in either wild-type or Fxrα−/− males fed a BA-diet (Fig. 4b). These differences should be due to the fact that in contrast to previous study by Baptissart *et al*. we have used alternation of diets due to the hyper-sensibility of *Fxrα*−/− mice to BA-diet.

### Germ cell apoptosis is correlated with altered expression of meiotic genes

To decipher the involved mechanisms in the observed phenotype of testis of Fxrα−/− mice, we first analyzed the mRNA accumulation of genes involved in spermatogenesis. No effect was observed for genes specific of pre-meiotic spermatogonia such as the *promyelocytic leukaemia zinc finger (Plzf* ) (Fig. 4c). In contrast in Fxrα−/− mice BA exposure led to a decreased mRNA accumulation of two key genes of the meiotic process: the *Stimulated by retinoic acid gene-8 (Stra-8*) and *DNA meiotic recombinase-1 (Dmc-1*) (Fig. 4c). These results support the idea that BA-diet altered the entry and/or the progression into meiosis. In that line the mRNA accumulation of post-meiotic gene *Tpn-1* (Transition protein-1) was decreased only in Fxrα−/− males fed BA-diet (Fig. 4c).

### PXR and CAR signaling pathways are modulated in a tissue dependent manner in response to altered BA homeostasis

**Figure 5:**
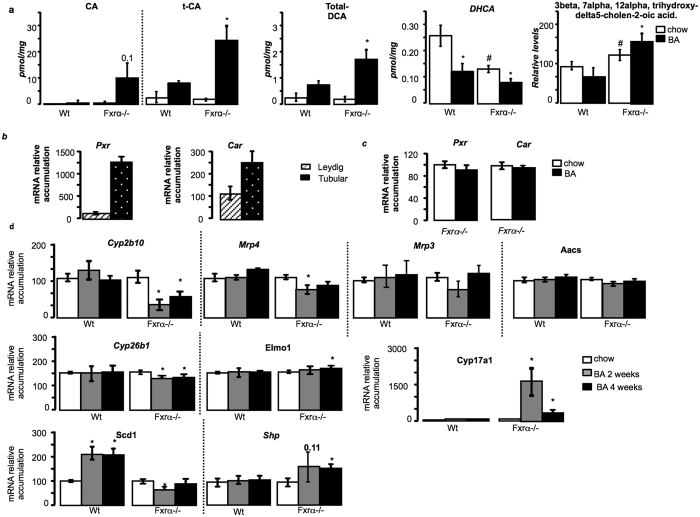
Fxrα is a gatekeeper of BA homeostasis in liver and testis in the context of BA exposure. (**a**) Testicular concentrations of BAs (CA, tCA, DCA), the intermediate BA metabolites DHCA and 3β, 7α, 12 α tri-OH-5-cholestenic acid, in testis of Wt and Fxrα−/− males fed a control or BA-diet for 2 weeks (n = 5 per group). (**b**) mRNA accumulations of *Pxr* and *Car* in tubular compartment or purified Leydig cells of C57Bl6 males. (**c**) Testicular mRNA accumulation of *Pxr* and *Car* normalized to *β*-*actin* mRNA levels in wild-type and Fxrα−/− mice fed a control or BA-diet for 1 month (n = 6–10 per group). (**d**) Testicular mRNA expression of *Cyp2b10, Mrp4, Mrp3*, Aacs, Cyp26b1, Elmo1, Cyp17a1, Scd1 and Shp normalized to *β*-*actin* mRNA levels in wild-type and Fxrα−/− adult mice fed a control or BA-diet for 2 or 4 weeks (n = 6–10 per group). In all panels, control diet groups were arbitrarily fixed at 100%. In all panels, data are expressed as means ± standard error of the mean. Statistical analysis: *P < 0.05.

Regarding the phenotype observed, and the lack of modulation of TGR5 signaling pathways, the next objective was to decipher the mechanisms how BA could act in Fxrα−/− testis. Interestingly, some BAs have been demonstrated to be modulators of PXR and/or CAR activities. Thus, LCA is an activator of Pregnane-X-Receptor (PXR; NR1I2); whereas the transcritpional activity of the Constitutive Androstan Receptor (CAR; NR1I3) is repressed by several bile acids, among which CA, tauro-CA and conjugated form of DCA^16^. No modulation was observed among groups and treatment regarding LCA levels (Fig. S1c). Consistently with CAR modulation, Fxrα−/− mice fed BA-diet showed higher concentrations of CA, tCA, and DCA compared to Fxrα−/− mice under chow diet, whereas no effect was observed in wild-type males (Fig. 5a). In addition, abnormal expression of genes involved in BA-synthesis pathways have been associated with the production of intermediate metabolites that have been identified to modulate the activity of the nuclear receptors CAR and PXR. Indeed, C*yp27a1* deficiency in mice (*Cyp27a1*−/−) was shown to be associated with an accumulation of BA precursors that are PXR agonists^17^, and mutation of *Hsd3b7* was associated with the production of intermediates defined as CAR inverse agonists^18^. In the present study, the altered expression levels of these genes in liver and in testis supports the idea of the production of intermediate metabolites of BA that could modulate PXR and/or CAR activities (Fig. 2d,e). In that line, the testicular levels of the late BA intermediate metabolite, DHCA, were decreased in Fxrα−/− compared to Wt and were specifically decreased in both Wt and Fxrα−/− mice fed a BA-diet (Fig. 5a). These data sustained the idea of the alteration of the intra-testicular BA synthesis and consequently accumulation of early intermediates. In that line, regarding literature more specific intermediates were analyzed with a higher level of 3β, 7α, 12 α tri-OH-5-cholestenic acid in Fxrα−/− males fed BA-diet compared to chow-diet group (Fig. 5a). Note that higher level of 3β, 7α, 12 α tri-OH-5-cholestenic acid was also observed in Fxrα−/− compared to wild-type animals (Fig. 5a).

All these data suggest that PXR and/or CAR signaling pathways should be involved in the phenotype observed in Fxrα−/− males fed BA-diet. We then validated this hypothesis first by showing that *Pxr* and *Car* are expressed within the testis. They are mainly detected in tubular compartment of testis (Fig. 5b). However, no effect was observed on their expression levels following BA-exposure in *Fxrα*−/− males (Fig. 5c). To decipher if their activities could be modulated we analyzed the expression of target genes. In testis, no effect was observed on the mRNA accumulation of PXR target genes such as *Acetoacetyl-CoA Synthetase (Aacs), Multidrug resistance-associated protein 3 (Mrp3; Abcc3)* after exposure to BA-diet (Fig. 5d). Interestingly, the testicular mRNA accumulations of *Cyp2b10* and Mrp4 were decreased in Fxrα−/− males exposed 2 or 4-weeks to BA-diet compared to chow diet animals (Fig. 5d). These data support the idea that CAR signaling pathway must be affected in the *Fxrα*−/− males fed a BA-diet. In order to comfort such hypothesis, we analyzed the expression of other genes described in the liver to be CAR specific versus PXR^19^. It is interesting to note that among them Cui *et al*. identified *Cyp8b1, Hsd3b7* and *Cyp27a1*, which were all affected in the Fxrα−/− mice fed BA-diet (Fig. 2e). In addition, data showed that *Cyp26b1, Elmo1, Cyp17a1, Scd1 and Shp* were affected by BA-diet in FXRα−/− males (Fig. 5d). This is consistent with the lower expression of *Hsd3b7* in these males as HSD3B7 mutation was demonstrated to be associated with the production of CAR inverse-Agonists.

Here the deregulation of *Shp* expression specifically in Fxrα−/− males fed BA-diet is of interest regarding the observed phenotype. Indeed, a previous link was made between SHP and the meiotic process^20^, where SHP inhibits germ cell differentiation through the inhibition of *Stra8* expression.

### CAR-signaling pathways participate to the testicular impact of BA-diet in the Fxrα−/− context

**Figure 6:**
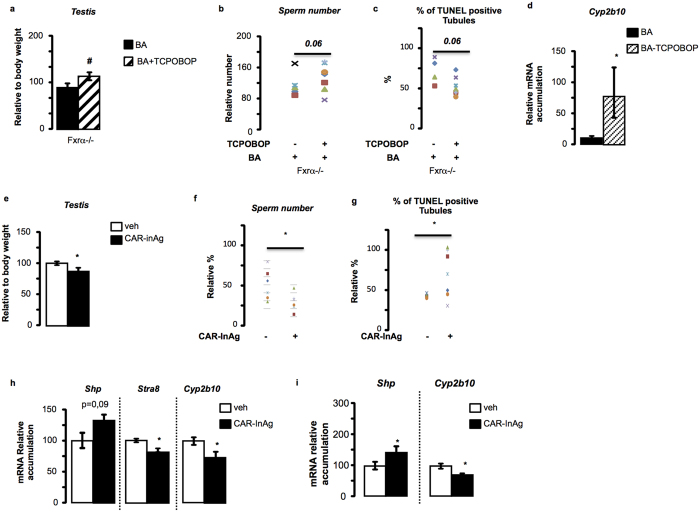
Bile acids act *via* CAR in the testis of Fxα−/− males. (**a**) Testis weights in Fxrα−/− mice treated with vehicle or TCPOBOP (6 mg/kg) in conjunction with BA-diet for 2 weeks. (**b**) Sperm count in Fxrα−/− mice treated with vehicle (DMSO) or TCPOBOP (6 mg/kg) in conjunction with BA-diet for 2 weeks. (**c**) Apoptosis in Fxrα−/− mice treated with vehicle or TCPOBOP (6 mg/kg) in conjunction with BA-diet for 2 weeks (n = 5–7 per group) analyzed by TUNEL staining. For the quantification of TUNEL analyses, the number of tubules with TUNEL-positive cells is indicated as the number of positive tubes per 100 seminiferous tubules (n = 5–7). (**d**) Testicular mRNA accumulation of *Cyp2b10* normalized to *β*-*actin* mRNA levels in Fxrα−/− mice treated with vehicle or TCPOBOP (6 mg/kg) in conjunction with BA-diet for 2 weeks (n = 5–7 per group). (**e**) Testis weights in C57Bl6J mice treated with vehicle or CAR-InAg for 2 weeks (n = 5–7 per group). (**f**) Sperm count in C57Bl6J mice treated with vehicle or CAR-InAg for 2 weeks (n = 5–7 per group). (**g**) Apoptosis in C57Bl6J mice treated with vehicle or CAR-InAg for 2 weeks analyzed by TUNEL staining. For the quantification of TUNEL analyses, the number of tubules with TUNEL-positive cells is indicated as the number of positive tubes per 100 seminiferous tubules (n = 5–7 per group). (**h**) Testicular mRNA accumulations of *Shp, Stra8 and Cyp2b10* normalized to *β*-*actin* mRNA levels in C57Bl6 males treated 2 weeks with CAR-InAg (30 mg/kg/day) or vehicle (DMSO) (n = 5–7 per group). (**i**) mRNA expression of *Shp and Cyp2b10* normalized to *β*-*actin* mRNA levels in GC1spg cell line treated 12 h or CAR-InAg (10^−6^ M) or vehicle (DMSO 1/1000). In all panels, data are expressed as means ± standard error of the mean. Statistical analysis: *P < 0.05.

To analyze *in vivo* the role of CAR signaling pathways in the phenotype observed in Fxrα−/− mice fed BA-diet, we performed an experiment where Fxrα−/− mice fed BA-diet were co-exposed to a CAR agonist (TCPOBOP). The goal was to define if it could reverse or at least attenuate the testicular phenotype observed in Fxrα−/− mice fed BA-diet. Interestingly, co-administration of TCPOBOP reversed the effect of BA-diet on testicular weights (Fig. 6a), on spermatozoa number (Fig. 6b), on pro-apoptotic effect of BA-diet (Fig. 6c), as well as on the impact on *Cyp2b10* mRNA accumulation (Fig. 6d).

Then, to comfort these results on the potential involvement of CAR signaling pathways in the observed testicular phenotypes in Fxrα−/− males exposed to BA-diet, we exposed C57Bl6 mice to a CAR inverse agonist (CAR-InAg); e.g. 5α-androstan3β-ol^21^. In addition, to exclude the involvement of PXR, the experiment was performed using the PXR agonist PCN. No effect of PCN was noticed for all the testicular parameters studied (Fig. S2a to S2d). The efficiency of PCN exposure was validated with the hepatic increased of *Mrp3* and *Cyp2b10* mRNA accumulation after 10-days of PCN treatment (Fig. S2e). This suggests that even if PXR agonists could be produced from the decreased expression of *Cyp27a1* in testis of *Fxrα*−/− mice fed BA-diet, and if PXR might be involved in liver alteration, its signaling might not be involved in the observed testicular phenotype.

C57Bl6 mice treated for 2-weeks with a CAR inverse agonist showed a decrease of testicular weights compared to vehicle group (Fig. 6e), which was correlated with a decrease of sperm production (Fig. 6f). This lower sperm production was associated with an increased rate of apoptotic germ cells following CAR-InvAg (Fig. 6g). At the molecular level, the exposure to the CAR-InAg led to an increase of *Shp* mRNA accumulation and a lower mRNA accumulation of *Stra8* and *Cyp2b10* (Fig. 6h). These data allowed us determining that exposure of C57Bl6J mice to CAR-InAg was able to strictly reproduce the testicular phenotype observed in *Fxrα*−/− mice fed BA-diet.

The impact of CAR signaling pathways within the germ cells was validated using the GC1spg germ cell line. Indeed, exposure of cell line to CAR-InvAg was able to reproduce the same molecular signature with an increase of *Shp* and a decrease of *Cyp2b10* accumulations (Fig. 6i).

## Discussion

**Figure 7:**
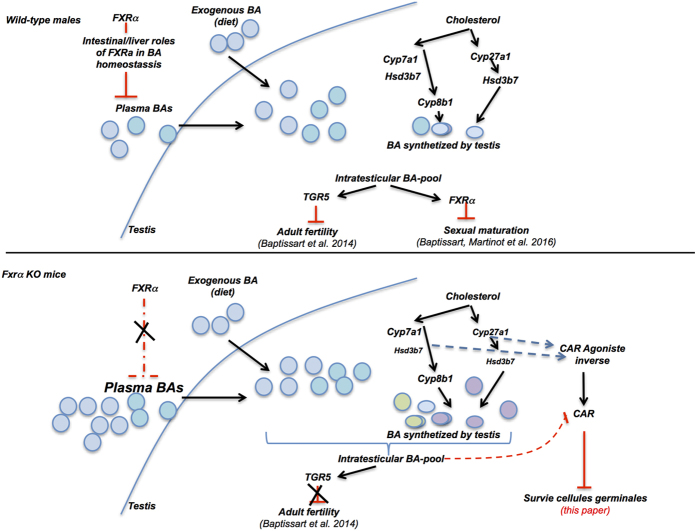
Representative scheme of the impact of BA-diet depending on FXR genotypes in testicular physiology.

Abnormal BA homeostasis has been associated with several diseases such as cholestasis or colon cancer. FXRα is a gatekeeper of BA homeostasis and plays major roles on physiology and pathophysiology. The identification of such particular regulation of BA homeostasis in different tissues by FXRα is critical, as several mutations have been reported in humans. Indeed, variants of FXRα have been correlated with intrahepatic cholestasis of pregnancy^22^. In addition, heterozygous variant of FXRα has been reported in one patient with infantile cholestasis^23^ and homozygous loss of FXRα function was associated with severe neonatal cholestasis^12^. This highlights the need to better define the numerous impacts of FXRα signaling pathways on physiology and related diseases. The present report suggests a new important aspect of BA homeostasis and their intrinsic synthesis within particular organs and its regulation by FXRα. This is of importance as our results defined in testis (Fig. 7) could thus be extrapolated to other organs expressing enzymes involved in BA synthesis.

In the liver, the lack of Fxrα led to a lower accumulation of Cyp27a1 and the mRNA accumulation of *Cyp8b1, Cyp7b1* and *Hsd3b7* were downregulated in the liver of *Fxrα*−/− males treated with BA-diet compared to Fxrα−/− males treated with control-diet (Fig. 2d). Such abnormalities support the idea of the production of intermediate metabolites of BA and thus the modulation of PXR and/or CAR activities. Our results sustained the hypothesis of the over-activation of PXR in the liver of *Fxrα*−/− mice fed BA-diet with the increased mRNA accumulation of PXR target genes such as *Sult2a1, Cyp2b10* and *Cyp3a11*. (Fig. S3) Consistently several studies analyzed the potential involvement of PXR in the hepatic regulation of genes involved in detoxification^24^.

Within testis, the present data show that FXRα is a gatekeeper of local bile acid homeostasis. Indeed, our data define an intra-testicular production of BA as demonstrated by BA measurement in medium of primary culture of Leydig cells and the mRNA and protein accumulations of enzymes involved in BA synthesis in whole testis. This intra-testicular BA synthesis is regulated by FXRα. The role of FXRα in testicular BA homeostasis is exacerbated by the alteration of BA composition and concentration in Fxrα−/− mice fed BA-diet. Within testis BA metabolism must be complex as depending on cell types studied the enzymes expressed are not the same. It appears that within the Leydig cells, only the alternative pathway could be performed; whereas tubular compartment might be able to achieve both classical and alternative pathways. These expression patterns might be associated with the production of specific BAs and the activation of different BA signaling pathways. In an integrative point of view, it suggests that BAs produced by the testis might be involved in a local control of testicular physiology.

In addition, our results sustain the idea of the altered BA homeostasis and of the production of intermediate BA metabolites within the testis, which led to the modulation of the CAR signaling pathways. These results were validated using co-exposure to TCPOBOP and BA-diet in *Fxrα*−/− mice and using a specific CAR inverse agonist in wild-type males that respectively counteracted or reproduced the phenotype observed in *Fxrα*−/− males fed BA-diet. These data open interesting perspectives beyond the area of reproductive field to better define how BA homeostasis might contribute to physiological or pathophysiological conditions via the modulation of CAR activity. In that line, the administration of a CAR-InAg was able to reproduce the testicular phenotype observed in Fxrα−/− males fed BA-diet (modulations of *Shp, Stra8* and *Cyp2b10* expression and increased rate of apoptotic germ cells). This sustains the hypothesis that the down-regulation of genes involved in BA synthesis must activate alternative pathway *via* CAR. Such deregulation of the pathways could find some relevance in cases of human pathologies such as in rare diseases of the deficit of primary bile acid synthesis with Hsd3b7 mutation. This pathology is treated with cholic acid. It is a critical treatment which allows maintaining the patients alive without liver transplantation. However, so far, no study has been conducted on these children treated with CA regarding their quality of life.

To our knowledge, few have been done regarding the pathophysiological consequences of CAR inverse agonist. Thus it cannot be excluded that the modulation of the CAR pathway due to the production of intermediate metabolites of BAs could be relevant in physiology and/or pathophysiology of other organs. In that line, regarding BA synthesis pathways, *Cyp27a1* is quite ubiquitous^25^ and *Cyp7b1* was previously described in several other organs such as liver, lungs, kidneys, brain and reproductive tract^26^. *Cyp7a1* and *Cyp8b1* were also reported in human ovary^27^. This is of importance as our results could thus be extrapolated to other organs expressing enzymes involved in BA synthesis. In that line, it might be important to re-screen in many tissues the expression levels of genes involved in BA synthesis as well as their potential cellular co-expression with PXR, CAR and/or FXRα in different specific cell types of the targeted organs. The present work is thus opening new field that could be enlarged to other organs and must help to better characterize the involvement of BA homeostasis in physiological and pathophysiological conditions.

## Methods

### Ethics statement

This study was conducted in accordance with the current regulations and standards approved by the Animal Care Committee (C2E2A Auvergne; protocol CE 07-12).

Animals: C57Bl/6J were purchased from the Charles River Laboratories (L’Arbresle, France); FXRα −/− mice have been previously described^5^,^6^,^28^. Mice used in this study were maintained on housed in temperature-controlled rooms with 12 hours light/dark cycles. Mice had *ad libitum* access to food and water. Nine-week- old mice were fed D04 diet (control) or D04 diet supplemented with 0.2% cholic acid (BA-diet) (SAFE, Augy, France). As FXRα−/− mice are quite sensitive to BA-diet, they were fed 5 days with CA-diet and 2 following days with the control diet. This sequence was repeated until sacrifice.

C57Bl6 mice were injected once a day for 15 days with 50 μl intraperitoneally with the PXR agonist PCN (50 mg/kg), or the CAR-InvAg 5α-androstan-3β-ol (30mg/kg), TCPOBOP (6 mg/kg) or vehicle (DMSO).

Leydig purified cells and tubular compartment were generated as described in previous study^29^.

### Histology

After exposure, the testes were collected, formalin-fixed and embedded in paraffin, and 5 μm-thick sections were prepared and stained with hematoxylin/eosin (n = 6–10 animals per group).

For the analysis of the blood-testis barrier integrity, 15 μl of EZ-Link Sulfo-NHS-LC-Biotin (7.5 mg/ml) was injected intra-peritoneally (200 μl) or an intratesticular (15 μl) injection of 0.6 mg of cholyl-lysyl-fluorescein (BD Bioscience, Le Pont de Claix, France)^5^. Thirty min after injection, the testes were harvested, formalin-fixed and embedded in paraffin, and 5 μm-thick sections were prepared.

### TUNEL analysis

TUNEL experiments were performed as previously described^30^ on 5 μm of testis fixed in PFA 4%. In each testis, at least 100 random seminiferous tubules were counted. The results are expressed as the number of tubules with either spermatocytes or spermatids TUNEL-positive per 100 seminiferous tubules.

### Endocrine Investigations

Testosterone was extracted from testis as previously described^30^. Intra-testicular and plasma testosterone levels were measured using a commercial kit (Diagnostic Biochem, London, Canada).

### Bile acid measurements

The measurements of total BAs were performed as previously described^13^ and using ELISA assays as recommended by manufacturer (Crystal Chem, Inc. Cat# 80470).

Bile acid pool composition was established as previously described^5^

### Real-Time RT-PCR

RNA from liver or testis samples was isolated using Nucleospin RNA L (Macherey-nagel, Hoerdt, France). cDNA was synthesized from total RNA with the MMLV reverse transcriptase and random hexamer primers (Promega, Charbonnière Les Bains, France). The real-time PCR measurement of individual cDNAs was performed using SYBR green dye (Master mix Plus for SYBR Assay, Eurogentec, Angers, France) to measure duplex DNA formation with the Eppendorf-Realplex system. For each organs, standard curves were generated with pools of testis cDNA from animals with different genotypes and/or treatments. The results were analyzed using the ΔΔct method.

Some of the primers were used in previous studies. Actin, Tgr5, Tbx2, Cx43, Sult2a1 and *Cyp3a11*^5^; *Stra8, Dmc1* and *Shp*^18^,^27^; *Cyp2b10, Cyp7a1, Cyp8b1, Cyp27a1, Mrp3, Mrp4, Mrp2, Pxr* and *Car*^31^; Rhox5 and *Osp*^13^. The sequences of the other primers are given in supplemental information in Table S1.

Cell Studies. GC1-spg cells were used as previously described^18^. Cells were treated for 12 hours with vehicle (DMSO, 1/1000) or CAR InAg (10^−5^ M; Sigma-Aldrich, St. Louis, MO), and messenger RNA (mRNA) or protein extractions were performed.

### Primary culture of Leydig cells

Purification of Leydig cells and cell culture were performed as previously described^32^. Briefly, testes from 90-day-old male mice were decapsulated and incubated for 20 min at 33 °C in Dulbecco’s modified Eagle’s medium (DMEM)/Ham’s F12 (1:1), transferrin (5 μg/ml), insulin (4 μg/ml) and vitamin E (0·2 μg/ml) medium containing collagenase (0·8 mg/ml) (Life Technologies, Invitrogen, Cergy-Pontoise, France). Extracts were collected by centrifugation for 10 min at 200 g and the pellet was resuspended in fresh medium. Following two successive sedimentations, the supernatant containing Leydig cells was centrifuged and the pellet was resuspended in fresh medium at a final concentration of 10^7^ cells/ml. Five milliliters of the suspension was layered on top of a discontinuous Percoll gradient (four layers from 21 to 60%) prepared from a stock solution (90% Percoll:10%Ham’s F10 (10X), 20 mM Hepes, 140 mMNaHCO3, pH 7·4) diluted with DMEM/Ham’sF12. After centrifugation for 30 min at 2000 g at 4 °C, purified Leydig cells were collected at the 60–34% layer interface, diluted in fresh medium and washed twice. Cells were seeded in fetal calf serum-pretreated six-well plates (2.0^6^ cells/well) in DMEM/Ham’s F12 (1:1), transferrin (5 μg/ml), insulin (4 μg/ml) and vitamin E (0·2 μg/ml) medium.

## Additional Information

**How to cite this article**: Martinot, E. *et al*. Bile acid homeostasis controls CAR signaling pathways in mouse testis through FXRalpha. *Sci. Rep.* **7**, 42182; doi: 10.1038/srep42182 (2017).

**Publisher's note:** Springer Nature remains neutral with regard to jurisdictional claims in published maps and institutional affiliations.

## Supplementary Material

Supplemental Information

## References

[b1] Neale G., Lewis B., Weaver V. & Panveliwalla D. Serum bile acids in liver disease. Gut 12, 145–152 (1971). 5548561 10.1136/gut.12.2.145 PMC1411536

[b2] Houten S. M., Volle D. H., Cummins C. L., Mangelsdorf D. J. & Auwerx J. *In vivo* imaging of farnesoid X receptor activity reveals the ileum as the primary bile acid signaling tissue. Mol. Endocrinol. 21, 1312–1323 (2007). 17426284 10.1210/me.2007-0113

[b3] Yamazaki M. . Perturbation of bile acid homeostasis is an early pathogenesis event of drug induced liver injury in rats. Toxicol. Appl. Pharmacol, doi: 10.1016/j.taap.2013.01.018 (2013). 23360887

[b4] Vítek L. & Haluzík M. The role of bile acids in metabolic regulation. J. Endocrinol. 228, R85–96 (2016). 26733603 10.1530/JOE-15-0469

[b5] Baptissart M. . Bile acids alter male fertility through G-protein-coupled bile acid receptor 1 signaling pathways in mice. Hepatology 60, 1054–1065 (2014). 24798773 10.1002/hep.27204

[b6] Baptissart M. . Bile acid-FXRα pathways regulate male sexual maturation in mice. Oncotarget, doi: 10.18632/oncotarget.7153 (2016). PMC4991395 26848619

[b7] Goodwin B. . A regulatory cascade of the nuclear receptors FXR, SHP-1, and LRH-1 represses bile acid biosynthesis. Mol. Cell 6, 517–526 (2000). 11030332 10.1016/s1097-2765(00)00051-4

[b8] Lu T. T. . Molecular basis for feedback regulation of bile acid synthesis by nuclear receptors. Mol. Cell 6, 507–515 (2000). 11030331 10.1016/s1097-2765(00)00050-2

[b9] Houten S. M., Watanabe M. & Auwerx J. Endocrine functions of bile acids. EMBO J. 25, 1419–1425 (2006). 16541101 10.1038/sj.emboj.7601049 PMC1440314

[b10] Fu T. . FXR Primes the Liver for Intestinal FGF15 Signaling by Transient Induction of β-Klotho. Mol. Endocrinol. 30, 92–103 (2016). 26505219 10.1210/me.2015-1226 PMC4695634

[b11] Modica S. . Selective activation of nuclear bile acid receptor FXR in the intestine protects mice against cholestasis. Gastroenterology 142, 355–365–4 (2012). 22057115 10.1053/j.gastro.2011.10.028

[b12] Gomez-Ospina N. . Mutations in the nuclear bile acid receptor FXR cause progressive familial intrahepatic cholestasis. Nat Commun 7, 10713 (2016). 26888176 10.1038/ncomms10713 PMC4759630

[b13] Vega A. . Bile Acid Alters Male Mouse Fertility in Metabolic Syndrome Context. PLoS ONE 10, e0139946 (2015). 26439743 10.1371/journal.pone.0139946 PMC4595338

[b14] Sinal C. J. . Targeted disruption of the nuclear receptor FXR/BAR impairs bile acid and lipid homeostasis. Cell 102, 731–744 (2000). 11030617 10.1016/s0092-8674(00)00062-3

[b15] Sharpe R. M., Donachie K. & Cooper I. Re-evaluation of the intratesticular level of testosterone required for quantitative maintenance of spermatogenesis in the rat. J. Endocrinol. 117, 19–26 (1988). 2833551 10.1677/joe.0.1170019

[b16] Moore L. B. . Pregnane X receptor (PXR), constitutive androstane receptor (CAR), and benzoate X receptor (BXR) define three pharmacologically distinct classes of nuclear receptors. Mol. Endocrinol. 16, 977–986 (2002). 11981033 10.1210/mend.16.5.0828

[b17] Goodwin B. . Identification of bile acid precursors as endogenous ligands for the nuclear xenobiotic pregnane X receptor. Proc. Natl. Acad. Sci. USA 100, 223–228 (2003). 12509506 10.1073/pnas.0237082100 PMC140933

[b18] Cui J. Y. & Klaassen C. D. RNA-Seq reveals common and unique PXR- and CAR-target gene signatures in the mouse liver transcriptome. Biochim. Biophys. Acta 1859, 1198–1217 (2016). 27113289 10.1016/j.bbagrm.2016.04.010 PMC5552365

[b19] Gioiello A. . Synthesis of atypical bile acids for use as investigative tools for the genetic defect of 3β-hydroxy-Δ(5)-C27-steroid oxidoreductase deficiency. J. Steroid Biochem. Mol. Biol. 144 Pt B, 348–360 (2014). 24954360 10.1016/j.jsbmb.2014.06.008

[b20] Volle D. H. . The orphan nuclear receptor small heterodimer partner mediates male infertility induced by diethylstilbestrol in mice. J. Clin. Invest. 119, 3752–3764 (2009). 19884658 10.1172/JCI38521 PMC2786790

[b21] Forman B. M. . Androstane metabolites bind to and deactivate the nuclear receptor CAR-beta. Nature 395, 612–615 (1998). 9783588 10.1038/26996

[b22] Van Mil S. W. C. . Functional variants of the central bile acid sensor FXR identified in intrahepatic cholestasis of pregnancy. Gastroenterology 133, 507–516 (2007). 17681172 10.1053/j.gastro.2007.05.015

[b23] Chen X.-Q. . A novel heterozygous NR1H4 termination codon mutation in idiopathic infantile cholestasis. World J Pediatr 8, 67–71 (2012). 21633855 10.1007/s12519-011-0299-z

[b24] Teng S. & Piquette-Miller M. Hepatoprotective role of PXR activation and MRP3 in cholic acid-induced cholestasis. Br. J. Pharmacol. 151, 367–376 (2007). 17435798 10.1038/sj.bjp.0707235 PMC2013976

[b25] Lorbek G., Lewinska M. & Rozman D. Cytochrome P450s in the synthesis of cholesterol and bile acids--from mouse models to human diseases. FEBS J. 279, 1516–1533 (2012). 22111624 10.1111/j.1742-4658.2011.08432.x

[b26] Stiles A. R., McDonald J. G., Bauman D. R. & Russell D. W. CYP7B1: one cytochrome P450, two human genetic diseases, and multiple physiological functions. J. Biol. Chem. 284, 28485–28489 (2009). 19687010 10.1074/jbc.R109.042168 PMC2781391

[b27] Smith L. P. . The bile acid synthesis pathway is present and functional in the human ovary. PLoS ONE 4, e7333 (2009). 19806215 10.1371/journal.pone.0007333 PMC2752198

[b28] Milona A. . The normal mechanisms of pregnancy-induced liver growth are not maintained in mice lacking the bile acid sensor Fxr. Am. J. Physiol. Gastrointest. Liver Physiol. 298, G151–158 (2010). 19815629 10.1152/ajpgi.00336.2009 PMC2822506

[b29] Volle D. H. . The small heterodimer partner is a gonadal gatekeeper of sexual maturation in male mice. Genes Dev. 21, 303–315 (2007). 17289919 10.1101/gad.409307 PMC1785120

[b30] Volle D. H. . Multiple roles of the nuclear receptors for oxysterols liver X receptor to maintain male fertility. Mol. Endocrinol. 21, 1014–1027 (2007). 17341595 10.1210/me.2006-0277

[b31] Vega A. . Hepatotoxicity induced by neonatal exposure to diethylstilbestrol is maintained throughout adulthood via the nuclear receptor SHP. Expert Opin. Ther. Targets 18, 1367–1376 (2014). 25263461 10.1517/14728222.2014.964209

[b32] Baron S. . Hormonal and developmental regulation of the mouse aldose reductase-like gene akr1b7 expression in Leydig cells. J. Mol. Endocrinol. 31, 71–81 (2003). 12914526 10.1677/jme.0.0310071

