# Supplementary material for: Bile acid homeostasis controls CAR signaling pathways in mouse testis through FXRalpha
Source: Sci Rep. 2017 Feb 9;7:42182. doi: 10.1038/srep42182 (PMC5299845; doi:10.1038/srep42182)
Supplement: Supplemental Information [file srep42182-s1.pdf]

Bile acid homeostasis controls CAR signaling pathways in mouse testis through FXRalpha.

Emmanuelle MARTINOT[1,2,3,4], Marine BAPTISSART[1,2,3,4], Aurélie VEGA[1,2,3,4], Lauriane SEDES[1,2,3,4], Betty ROUAISNEL[1,2,3,4], Fred VAZ[5], Jean-Paul SARU[1,2,3,4], Angélique de HAZE[1,2,3,4], Silvère BARON[1,2,3,4], Françoise CAIRA[1,2,3,4], Claude BEAUDOIN[1,2,3,4] and David H. VOLLE[1,2,3,4*]

[1]INSERM U 1103, Génétique Reproduction et Développement (GReD), F-63170 Aubière, France

[2]Université Clermont Auvergne, GReD, F-63000 Clermont-Ferrand, F-63170 Aubière, France

[3]CNRS, UMR 6293, GReD, F-63170 Aubière, France

[4]Centre de Recherche en Nutrition Humaine d'Auvergne, F-63000 Clermont-Ferrand, France

[5] Academic Medical Center, University of Amsterdam, Lab. Genetic Metabolic Diseases, F0-224, P.O. Box 22700, 1100 DE Amsterdam, The Netherlands.

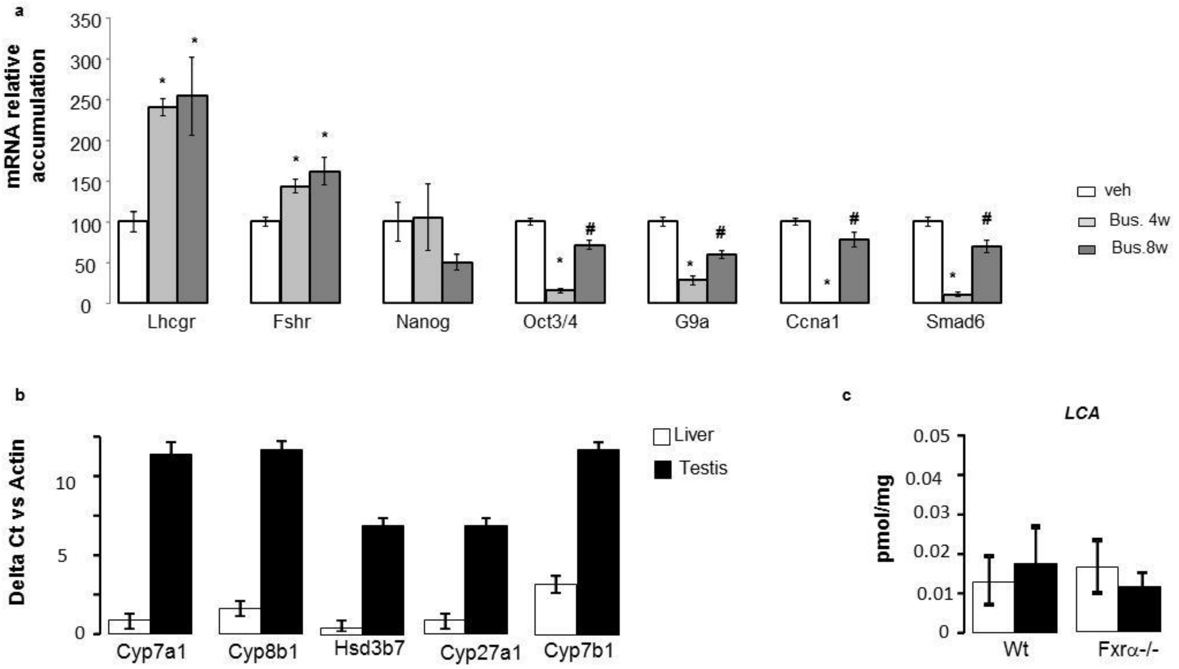

Fig. S1. a/ Testicular mRNA accumulation of *Lcgr, Fshr, Nanog, Oct3/4, G9a, Ccna1 and Smad6*, normalized to *β-actin* mRNA levels in whole testes of C57BL/6J mice treated with busulfan (20 mg/kg, one injection IP) at T0, 4, or 8 weeks (n = 8 per group). *Denotes significant difference from the T0 time point; #denotes significant difference from the 4-week time point ($P < 0.05$). **b/** Analysis of the relative liver and testicular levels of *Cyp7a1*, *Cyp8b1*, *Hsd3b7*, *Cyp27a1* and *Cyp7b1*. The relative levels are represented as delta-Ct versus β-actin. **c/** Testicular concentration of LCA in testis of Wt and Fxrα-/- males fed a control or BA-diet for 2 weeks (n = 5 per group).

In all panels, data are expressed as means ± standard error of the mean. Statistical analysis: *$P < 0.05$.

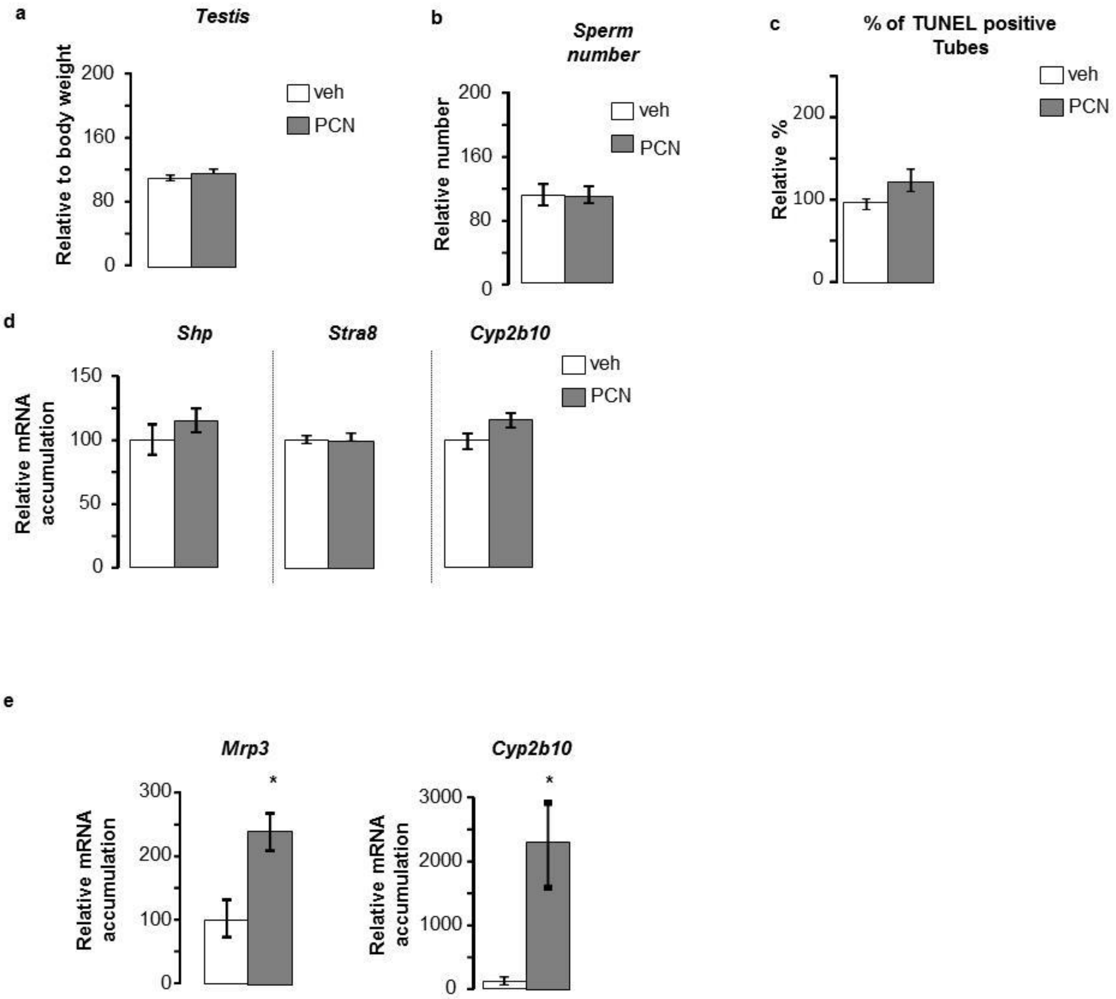

Fig. S2. a/ Testis weight normalized to body weight in wild-type C57Bl6 males treated 2 weeks with PCN (50mg/kg/day) or vehicle (DMSO) (n=6 to 7 per group). **b/** Spermatozoa count in the head of the epidydimis of C57Bl6 males treated 2 weeks with PCN (50mg/kg/day) or vehicle (DMSO) (n=6 to 7 per group). **c/** Quantification of TUNEL analyses in C57Bl6 males treated 2 weeks with PCN (50mg/kg/day) or vehicle (DMSO), the number of tubules with TUNEL-positive cells is indicated as the number of positive tubes per 100 seminiferous tubules (n = 6-7). **d/** Testicular mRNA accumulation of *Shp, Stra8 and Cyp2b10* normalized to *β-actin* mRNA levels in C57Bl6 males treated 2 weeks with PCN (50mg/kg/day) or vehicle (DMSO). Vehicle-treated mice were arbitrarily fixed at 100% (n=6 to 7 per group). **e/** Liver mRNA accumulation of *Mrp3 and Cyp2b10* normalized to *β-actin* mRNA levels in C57Bl6 males treated 2 weeks with PCN (50mg/kg/day) or vehicle (DMSO). Vehicle-treated mice were arbitrarily fixed at 100% (n=6 to 7 per group).

In all panels, data are expressed as means ± standard error of the mean. Statistical analysis: *$P < 0.05$.

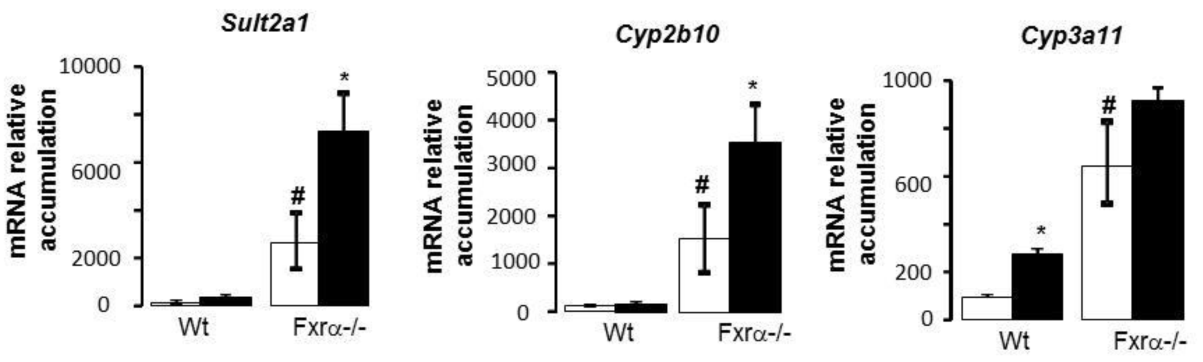

Fig. S3. Liver mRNA expression of *Sult2a1*, *Cyp2b10* and *Cyp3a11* normalized to *β-actin* mRNA levels in wild-type and Fxrα-/- adult mice fed a control or BA-diet (n = 6 -10 per group).

Data are expressed as means ± standard error of the mean. Statistical analysis: *$P < 0.05$.

Table S1

**PLZF**	**FW**	ACACAGACAGACCCATACTGG
	**REV**	TCACCTGTATGTGAGCGAAGG
**HSD3B7**	**FW**	ACCTGCACCTGAGTTCTTGG
	**REV**	CAATCACATTCTGTGTGCCC
**AACS**	**FW**	GTGGTGCTGATCCCCTATGT
	**REV**	CTTGCCAGGAAATCATCCAG
**TPN1**	**FW**	ACCAGCCGCAAGCTAAAGACTCAT
	**REV**	TCAGACTGGCATTTACCCACTCT
**TSX**	**FW**	ATGATGACACCGACGATCAGG
	**REV**	TCGGTACTGCTGACTCTGTCC
